# Inhibitory Effect of Mesenchymal Stem Cell Co-Culture on Erythroid Differentiation of K562 Cells Compared to The Control Group 

**DOI:** 10.22074/cellj.2016.4133

**Published:** 2016-12-21

**Authors:** Mahshid Saleh, Karim Shamsasanjan, Ali Akbari Movassaghpour, Parvin Akbarzadehlaleh, Zahra Molaeipour

**Affiliations:** 1 Hematology Oncology Research Center, Tabriz University of Medical Sciences, Tabriz, Iran; 2 Department of Pharmacutical Biotechnology, Tabriz University of Medical Sciences, Tabriz, Iran

**Keywords:** Mesenchymal Stem Cells, K562 Cells, Erythroid Differentiation

## Abstract

**Objective:**

Bone marrow mesenchymal stem cells (BMMSCs) reside in the bone marrow and control the process of hematopoiesis. They are an excellent instrument for regenerative treatment and co-culture with hematopoietic stem cells (HSCs).

**Materials and Methods:**

In this experimental study, K562 cell lines were either treated with butyric acid and co-cultured with MSCs, or cultivated in a conditioned medium from MSCs plus butyric acid for erythroid differentiation. We used the trypan blue dye exclusion assay to determine cell counts and viability in each group. For each group, we separately assessed erythroid differentiation of the K562 cell line with Giemsa stain under light microscopy, expression of specific markers of erythroid cells by flowcytometry, and erythroidspecific gene expressions by real-time polymerase chain reaction (RT-PCR).

**Results:**

There was enhandced erythroid differentiation of K562 cells with butyric acid compared to the K562 cell line co-cultured with MSCs and butyric acid. Erythroid differentiation of the K562 cell line cultivated in conditioned medium with butyric acid was higher than the K562 cell line co-cultured with MSCs and butyric acid, but less than K562 cell line treated with butyric acid only.

**Conclusion:**

Our results showed that MSCs significantly suppressed erythropoiesis. Therefore, MSCs would not be a suitable optimal treatment strategy for patients with erythroid leukemia.

## Introduction

Bone marrow mesenchymal stem cells (BMMSCs) reside in the bone marrow and support homing and differentiation of hematopoietic stem cells (HSCs) (1). MSCs are the spindle shaped plastic-adherent cells derived from bone marrow, adipose, and other tissue sources that have the capability to self-renew and undergo multipotent differentiation in vitro (2). BMMSCs are stem/progenitor cells that can self-renew and differentiate into osteoblasts, chondrocytes, adipocytes, and neural cells (3, 4). BMMSCs express unique surface markers-STRO-1, CD29, ecto-5´-endonucleotidase (CD73), CD90, endoglin (CD105), CD146, Octamer-4 (Oct4), stage-specific embryonic antigen-4 (SSEA4), GD2 ganglioside and CD271 (low affinity nerve growth factor receptor) (1, 5). It is commonly thought that BMMSCs do not express hematopoietic cell markers CD14 and CD34 (3, 6-9).

BMMSCs can preserve long term hematopoiesis *in vitro* and support the expansion and proliferation of hematopoietic colony forming cells in conjunction with added exogenous cytokines (10).

BMMSCs derived from adults produce signals for proliferation and differentiation of HSCs and their progenitors during direct cell-cell contact (11). These cells secrete cytokines and growth factors for HSC fate (12-14).

MSCs attach to HSCs by adhesion molecules such as N-cadherin and β integrins. Cytokines released by MSCs such as KIT-L, SDF-1, and Ang1 support the growth and differentiation of HSCs by binding to Kit, CXCR4 and Tie2 receptors. While HSCs are attached to MSCs, the expression of Notch ligands (Jagged and Delta-like) in MSCs is enhanced through the Wnt signaling pathway. Expression of Notch receptors in HSCs is enhanced by sonic hedgehog (Shh) in HSCs and MSCs (15) *in vitro* and decreases the repertoire of HSCs *in vivo* (16).

Wnt signaling pathway multilineage differentiation of MSCs and sustains them in an undifferentiated state (17). This signaling pathway has an essential role in self-renewal, survival, and proliferation of HSCs *in vitro* (18, 19). Erythropoiesis is a regular, continuous process in which HSCs proliferate and differentiate into mature red blood cells. The process is controlled by growth factors and cytokines. The most important growth factors are EPO and SCF (20, 21). The effects of MSCs on erythroid and myeloid differentiation may be due to specific cytokine lineage secreted by MSCs. Granulocyte colony-stimulating factor (G-CSF) and IL-6 secreted by MSCs are involved in the differentiation of HSCs *in vitro*. G-CSF is a key factor in myeloid differentiation and IL-6 in combination with SCF-induced proliferation of hematopoietic progenitor cells (22, 23).

MSCs are injected parallel to HSCs to enhance bone marrow transplantations (24, 25). However, the effects of MSCs on hematopoietic cell differentiation and possible molecular pathways are not well understood. Therefore, in this study we have investigated the effect of MSCs on erythroid differentiation of induced K562 cells as an erythroid differentiation model.

## Materials and Methods

### Cell culture 

#### K562 cell line culture

In this experimental study, the K562 cell line (Pasteur Institute, Iran) was cultivated in RPMI 1640 (Sigma-Aldrich, USA) medium supplemented with 10% fetal bovine serum (FBS, Gibco, UK), 100 U/ml streptomycin, 100 U/ml penicillin, and 0.2 mmol/L-glutamine (Gibco, UK) at 37˚C in a humidified incubator with a 5% CO_2_ atmosphere. Cells (1×10^5^cells per ml) were cultured for five days and passaged every two days to maintain a log phase growth.

#### Mesenchymal stem cell culture

BMMSCs were cultured in complete Dulbecco’s Modified Eagle’s Medium-low glucose (DMEM, Gibco, UK) supplemented with 10% FBS, 100 U/ml penicillin, 100 U/ml streptomycin, and 0.2 mmol/L glutamine at 37˚C in a humidified incubator with an atmosphere of 5% CO_2_. The cells were passaged until 60% confluency. The characterized BMMSCs were prepared from StemCell Technology Company, (Iran).

#### Assessment of cell viability

Cell counts and cell viability were determined by trypan blue dye (Sigma-Aldrich, USA) exclusion. Viable and nonviable cells were counted with a hemocytometer.

### Induction of erythroid differentiation (drug treatments)

Induction of K562 erythroid differentiation was carried out by 1 mM butyric acid (Orto, Germany) in K562 cells (1×10^5^/ml) for 24, 4, 8 or 72 hours.

### Culture of K562 cells with conditioned medium

K562 cells were cultured in conditioned medium with 1 mM butyric acid at 37˚C and 5% CO_2_. The conditioned medium was RPMI-1640 medium in which MSCs were cultured at 37˚C and 5% CO_2_ for 24 hours. The culture supernatant was collected and cultured with K562 cells.

### K562 cells co-cultured with mesenchymal stem cells

MSCs (1×10^4^/cm^2^) were cultured in a flask at 37˚C in a humidified incubator with 5% CO_2_. Once the cells reached 60% confluency, we added K562 cells (1×10^5^/ml) and 1 mM butyric acid. The co- culture was incubated at 37˚C in 5% CO_2_.

### Morphological assessment of erythroid differentiation

The cells were spread on collagen slides (slides suspended in collagen for 24 hours) and stained with Giemsa. Morphological assessment was visualized by light microscopy with oil immersion.

### Assessment of erythroid differentiation by flowcytometry

Erythroid differentiation of the cells was assessed by flowcytometry. In order to determine erythroid differentiation, we analyzed glycophorin A (GPA) on a surface of the differentiated cells by flowcytometry. The cells (1×10^5^) were harvested, washed twice with phosphate-buffered saline (PBS, Sigma-Aldrich, Germany) by centrifuging at 3500 g for 5 minutes at room temperature. Next, they were incubated with anti-GPA monoclonal antibody conjugated with FITC (Dako Cytomation, Denmark) at 4˚C for 30 minutes in the dark and analyzed with a FACSCalibur™ (Becton Dickinson, USA).

### RNA isolation and real-time polymerase chain reaction 

RNA was extracted by QIAzol (Qiagen, USA) from untreated and treated cultured K562 cells, after which cDNA was synthesized using 1 µg of total RNA and a cDNA synthesis kit (RevertAid First Strand cDNA Synthesis Kit, Fermentas, USA). Real-time polymerase chain reaction (RT-PCR) was used to detect expression of the erythroid geneby the One-Step Quantitech SyberGreen Real Time PCR kit (ABI) according to the manufacturer’s instructions. For RNA extraction, we collected a 106 K562 cell suspension after which one ml of QIAzol (Qiagen, USA) was added to the tubes and vortexed. The cells were incubated at -80˚C for 4-24 hours. Then, 200 µl chloroform was added to each tube. Samples were incubated at 4˚C for 5 minutes and centrifuged at 15000-20000 rpm at 4˚C for 30 minutes. After centrifugation, the supernatant was transferred to new microtubes and RNase-free isopropanol (100%) was added in the same volume. The microtubes were centrifuged at 18000 rpm at 4˚C for 20 minutes. The supernatant was removed and the RNA sediment was washed with a 75% ethanol solution. The washing step was repeated twice after which the supernatant was gently removed from the microtubes and left at room temperature for 10-30 minutes to dry. RNA was dissolved in 20 µl of Diethylpyrocarbonate (DEPC) water. Finally, the RNA concentration was measured with a spectrophotometer (Picodrop, Uk).

### cDNA synthesis

Briefly, for cDNA synthesis, we initially mixed 1 µg of RNA with 1 µl of the random hexamer primer and added nuclease-free water up to 12 µl. Subsequently the tubes were incubated at 65˚C for 5 minutes in a Thermocycler (Sensquest) after which the tubes were placed on ice (4˚C) and the following reagents were added: We placed the tubes in a Thermocycler and amplified cDNA with the following program of 5 minutes at 25˚C, 60 minutes at 42˚C, and 5 minutes at 70˚C.

**Table 1:** Reagents for cDNA synthesis


5X reaction buffer for reverse transcriptase	4 µl
RiboLock™ RNase inhibitor	(20 U/µl) 1 µl
dNTP Mix, 10 mM each	2 µl (one mM final concentration)
Revert Aid™ M-MuLV Reverse Transcriptase	(200 U/µl) 1 µl
RNA	1 µl
Random primers	1 µl
Total volume	20 µl


### Assessment of gene expression by real-time polymerase chain reaction

The relative expressions of specific genes in the erythrocytes were assessed by RT-PCR after a 48-hour incubation period. We used RT-PCR to determine expressions of the following genes: *AHSP, EPB42, FECH, ANK1, GLRX5, GATA2, NFE2, HBA2, HBB, HBG2, GYPA,* and *TFRC*. We added 5 µl of 2X qPCR/ RTD-PCR Master Mix E4 (SYBR Green AB Kit); 1 µl of an up-stream primer and 1 µl of a down-stream primer (maximum concentration: 200 nM, Metabion, Germany); 1 µl of cDNA (100 ng); and 2 µl of ddH_2_O to amplify the genes. Reactions were run in a RT- PCR device (AB Applied Biosystems, Stepone Realtime PCR) for 10 minutes at 95˚C, then 40 cycles as follows: 15 seconds at 95˚C and 60 seconds at 55-65˚C. GAPDH was the internal control.

**Table 2:** Primers for real-time polymerase chain reaction (RT-PCR)


Gene	Sequencing primer (5ˊ-3ˊ)

*AHSP*	F: GGTGGAGGACTGGATGAACTTC
	R: TCAGGAAGTCCCTGTACTTGGC
*EPB42*	F: ACCCAAGTGCTCCTAATGGAGG
	R: CCATCCTCACAGCACTTCCAGA
*FECH*	F: TCTTCTTGGACCGAGACCTCATG
	R: TCCAATCCTGCGGTACTGCTCT
*ANK1*	F: AAAACGGCTCCGTGTGGAAGGA
	R: GATGATTCGGCACACCCTCTTC
*GLRX5*	F: TCAGCAACGCCGTGGTGCAGA
	R: TTGAGGTACACTTGCGGGATGG
*GATA2*	F: CAGCAAGGCTCGTTCCTGTTCA
	R: ATGAGTGGTCGGTTCTGCCCAT
*NFE2*	F: GGAGAGATGGAACTGACTTGGC
	R: GAATCTGGGTGGATTGAGCAGG
*HBA2*	F: GACCTGCACGCGCACAAGCTT
	R: GCTCACAGAAGCCAGGAACTTG
*HBB*	F: CACCTTTGCCACACTGAGTGAG
	R: CCACTTTCTGATAGGCAGCCTG
*HBG2*	F: GGAAGATGCTGGAGGAGAAACC
	R: GTCAGCACCTTCTTGCCATGTG
*GYPA*	F: ATATGCAGCCACTCCTAGAGCTC
	R: CTGGTTCAGAGAAATGATGGGCA
*TFRC*	F: ATCGGTTGGTGCCACTGAATGG
	R: ACAACAGTGGGCTGGCAGAAAC
*GAPDH*	F: ACCCATCACCATCTTCCAGGAG
	R: GAAGGGGCGGAGATGATGAC


### Statistical analysis

Data were investigated by GraphPad Prism version 6.00 (GraphPad Software, Inc., La Jolla, CA). We used the student’s t test for single comparisons and one- way ANOVA for multi-group comparisons. The data were shown as mean ± S.D. P<0.01 was considered to be statistically significant.

## Results

### CD235a (GYPA) expressing cell population decreased in K562 cells co-cultured with mesenchymal stem cells

The K562 cell’s co-cultured with MSCs and those K562 cells cultivated in conditioned medium with 1 mM butyric acid had decreased expression of CD235a (GYPA), the erythroid differentiation marker, as assessed by flow cytometry after 48 hours. The K562 cells with butyric acid had higher expression of CD235a compared to those co-cultured with MSCs and K562 cells cultured in conditioned medium. There was higher expression of this marker in K562 cells cultured with conditioned medium compared to K562 cells co-cultured with MSCs. 

### Morphological study of K562 cells in different conditions 

K562 cells treated with 1 mM butyric acid and K562 cells co-cultured with MSCs and 1 mM butyric acid after a 48-hour incubation period were stained by Wright Giemsa.

### Gene expressions analysis of *AHSP, EPB42, FECH, ANK1, GLRX5, GATA2, NFE2, HBA2, HBB, HBG2, GYPA, TFRC* in the various cell groups

The butyric acid treated K562 cells had highly increase dexpressions of *GYPA, HBB, HBA2, NFE2,* and *FECH;* no significant increase in *ANK1, HBG2, GATA2, and AHSP* expressions; and decreased expressions of *TFRC* and *GLRX5*.

K562, cells co-cultured with MSCs had significantly increased expressions of *HBA2, GYPA, HBB, FECH,* and *NFE2*; no significant increase in expressions of *HBG2, GATA2, ANK1,* and *AHSP*; and decreased expressions of *TFRC* and *GLRX5*.

K562 cells cultured in conditioned medium had significantly increased expressions of *HBA2, NFE2, GLRX5, HBB,* and *GYPA*; no significant increase in *FECH, ANK1, GATA2, HBG2,* and *AHSP*; and a decrease in *TFRC* expression. In this study, the *EPB42* gene was also examined, but showed no evidence of expression in any of the groups.

**Fig.1:**
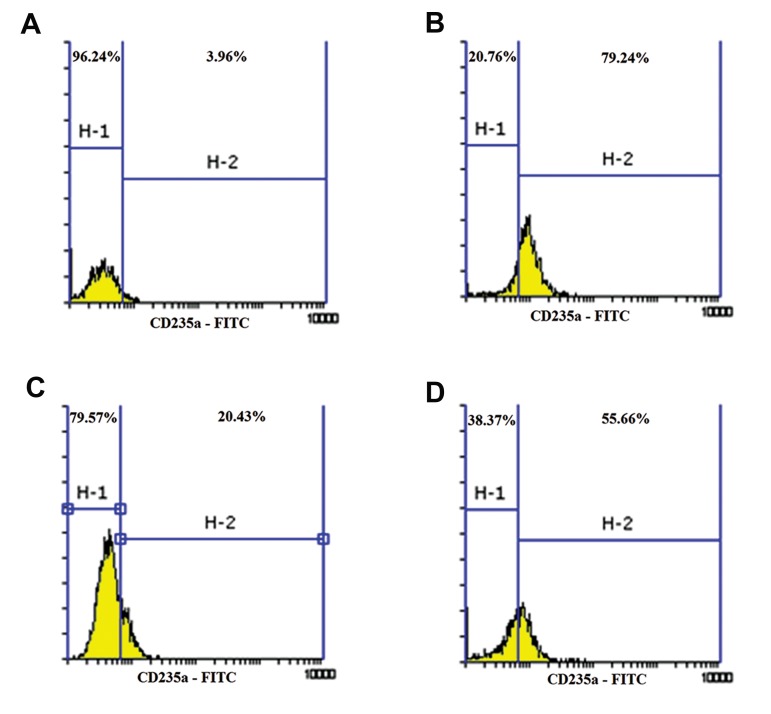
Flow cytometric analysis of erythroid marker differentiation after 48 hours. A. K562, B. K562+butyric acid (1 mM), C. K562+butyric acid (1 mM)+ mesenchymal stem cells (MSCs), and D. K562+butyric acid (1 mM) in conditioned medium.

**Fig.2:**
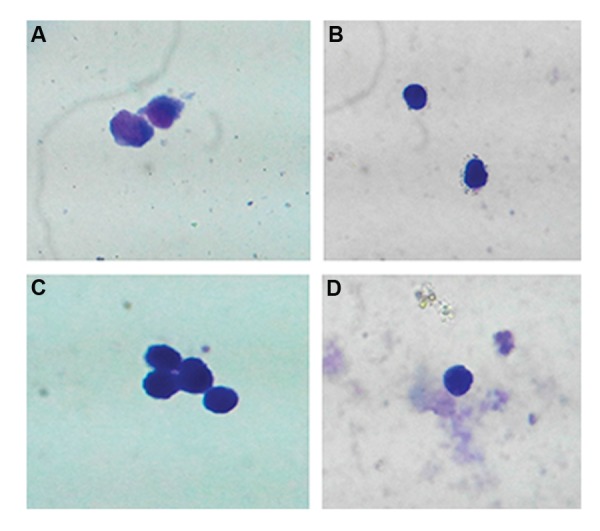
Microscopic analysis of k562 cell differentiation after 48 hours. A. Morphology of K562 cells (control), B. K562 cells treated with butyric acid (1 mM), C. Co-culture of K562 cells with mesenchymal stem cells (MSCs), and D. K562 cells plus butyric acid and MSCs (magnitude: ×100).

**Fig.3:**
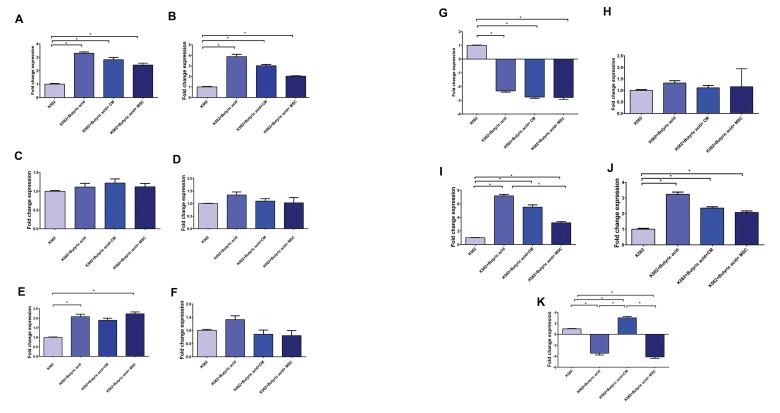
Gene expressions during differentiation of K562 cells after 48 hours. **A.** *GYPA*, **B.** *NFE2*, **C.** *AHSP*, **D.** *ANK1*, **E.** *FECH*, **F.** *GATA2*, **G.** *TFRC*, **H.** *HBG2*, **I.** *HBA2*, **J.** *HBB*, and **K.** *GLRX5*. *; Statistical significance level is P<0.01.

## Discussion

BMMSCs produce a variety of cytokines and extracellular matrix proteins to construct the bone marrow niche. Accordingly, the MSC feeder layer could be an appropriate model system to study HSCs *in vitro* (26). Studies have demonstrated that MSCs play a fundamental role in maintenance of stemness of HSCs in addition to their homing, proliferation, and differentiation (11, 27, 28). MSCs can influence various cell types, including leukemic cells (29, 30). Therefore, regarding the interaction of MSCs with leukemic cancer stem cells, these cells can be applied as an adjunctive therapy in leukemia treatment. 

Several researches on the co-culture of MSCs with HSCs confirmed that MSCs supported HSCs selfrenewal, proliferation, and differentiation (31-34). Fonseka et al. (35) showed that human umbilical cord blood-derived MSCs (hUCB-MSCs) were remarkably able to inhibit proliferation of K562 leukemic cells via cell-cell interactions. MSC sarrested the cell cycle of K562 cells at the G0/ G1 phase and prevented their entrance into the S phase. In this process, MSCs might secrete some anti-tumor cytokines such as interleukin-6 and -8.

Other studies demonstrated that BMMSCs from leukemia patients and normal individuals facilitated the proliferation and viability of K562 cells. Obvious suppression was seen in both cocultured MSCs and conditioned medium from MSCs (30). Han et al. (36) reported that the expansion of K562 cells drastically decreased when co-cultured with BMMSCs. Other studies showed that MSCs prevented K562 proliferation via the Wnt signaling pathway. They suggested that cell-cell contacts between MSCs and K562 cells induced the production of soluble factors such asdickkopf-1 (DKK1) which has been shown to suppress the Wnt signaling pathway and subsequently inhibit K562 expansion (37, 38). Other investigations demonstrated that soluble factors released by MSCs had more effect on inhibition of HSCs apoptosis and maintenance of their proliferation rather than direct cell-cell interaction. They showed that MSCs affected myeloid differentiation rather than erythropoiesis. Although there was an increased differentiation of myeloid cells, there were more erythroid cells in the control group (K562 cultured without MSCs). This implied that MSCs might have a supportive effect on erythroid differentiation (31).

Many studies have examined the co-culture of K562 cells with MSCs and the effect of MSCs on proliferation and apoptosis of this cell line. In this study, we investigated the effect of MSCs on erythroid differentiation of K562 cells in an induced situation.

We used RT-PCR to analyze the erythroidspecific gene expressions in K562 cells under three conditions: K562 cells treated with butyric acid; K562 cells co-cultured with MSCs and butyric acid; and K562 cells cultivated in conditioned medium from MSCs with butyric acid. Erythroidspecific genes analyzed included: *AHSP, EPB42, FECH, ANK1, GLRX5, GATA2, NFE2, HBA2, HBB, HBG2, GYPA,* and *TFRC*.

*HBA* and *HBB* are expressed in mature erythroid cells; these genes are essential for producing hemoglobin A by encoding α and β globins (39). We have shown that MSCs inhibited expressions of these genes. The transcription factors nuclear factor-erythroid 2 (*NFE2*) is an essential regulator of erythroid specific gene expression, composed of two subunits (p45 and p18). P54 is expressed in mature erythroid cells and other differentiated hematopoietic cell lines such as granulocytes, megakaryocytes, and mast cells (40). *NFE2* was suppressed by MSCs. Other erythroid specific genes did not show significant expressions. Hence, additional research is necessary to analyze these genes.

We found that MSCs decreased the differential effect of butyric acid on K562 cells. Our results demonstrated that K562 cells co-cultured with MSCs had less erythroid differentiation than K562 cells, which were cultured with conditioned medium. Soluble factors secreted by MSCs appeared to be less effective than cell-cell interactions. Various cytokines secreted by MSCs (IL-6 and G-CSF) have been shown to participate in differentiation and hematopoiesis of HSCs in vitro (23). It seems that these cytokines may play a critical role in erythroid differentiation of the K562 cell line. 

## Conclusion

Several studies researched the effect MSCs on proliferation, expansion, and apoptosis when co-cultured with K562 cells. Results of these studies were varied and sometimes contradictory. In the current study, MSCs have decreased erythroid differentiation of K562 cells. Additionally, our data demonstrated that cell-cell contact between MSCs and K562 cells suppressed erythroid differentiation. Although soluble factors secreted by MSCs might have few effects on erythroid differentiation, conditioned medium decreased the differential effect of butyric acid on K562 cells; this reduction was not less than that observed in cell-cell contact. Additional studies should be conducted in order to determine the cellular mechanisms of the effect of MSCs on differentiation of K562 cells.
